# Second Generation Long-Acting Injectable Antipsychotics in Africa: About a Case

**DOI:** 10.1192/j.eurpsy.2023.1737

**Published:** 2023-07-19

**Authors:** J. Galvañ, F. R. Nguepy-Keubo

**Affiliations:** 1 Department of Child and Adolescent Psychiatry, Institute of Psychiatry and Mental Health Hospital General Universitario Gregorio Marañón, Madrid, Spain; 2 Hôpital Saint Vincent de Paul, Dschang, Cameroon

## Abstract

**Introduction:**

Schizophrenia affects people worldwide. In Europe, the advantages of second-generation and long-acting injectable antipsychotics (SG-LAIs) are known and used, supported by scientific evidence. However, somewhere there is limited evidence on this topic.

**Objectives:**

Highlight the improvements in antipsychotic treatment and raise awareness of the scar between Europe and Africa, showing the results of the evidence with a case of cooperation with Cameroon.

**Methods:**

About a case of a 42-year-old Cameroonian woman with 25 years of schizophrenia, treated with first-generation antipsychotic polypharmacy (APP) oral and depot, with several psychotic relapses, disorganized behaviors, motor and cognitive impairment and isolation (telemedicine consultation received through a NGO platform).

A search on PubMed was performed, selecting two systematic reviews including “antipsychotic” AND “Africa”, one systematic review for SGAs and four reviews for LAIs.

**Results:**

Seven articles were reviewed, finding that APP use is highly prevalent in Africa with a lack of research on this, SGAs show an improved safety and tolerability profile and LAIs are among the most effective treatments in psychiatry improving adherence and overall patient outcomes.

In our case, we recommend progressively adjusting treatment to SG-LAI monotherapy, visiting the patient six months later in Cameroon, observing sustained stability of positive symptoms with an improvement of negative symptoms and good adherence and tolerability to treatment without extrapyramidal effects.

**Conclusions:**

Our case is an example of the evidence that supports the improvement that SG-LAIs represent in psychiatric treatment and how international cooperation can help bridge the gap between Africa and Europe. Nevertheless, more research is needed to build bridges.

**Disclosure of Interest:**

None Declared

